# Supplementary material for: Assessment of Diet Quality in Chilean Urban Population through the Alternate Healthy Eating Index 2010: A Cross-Sectional Study
Source: Nutrients. 2019 Apr 20;11(4):891. doi: 10.3390/nu11040891 (PMC6521181; doi:10.3390/nu11040891)
Supplement: Supplementary file 1 [file nutrients-11-00891-s001.pdf]

Article

Assessment of Diet Quality in Chilean Urban Population through the Alternate Healthy Eating Index 2010: A Cross-Sectional Study

Victoria Pinto [1], Leslie Landaeta-Díaz [2], Oscar Castillo [3], Luis Villarroel [4], Attilio Rigotti [1,5], Guadalupe Echeverría [1,5,*] and ELANS Study Group

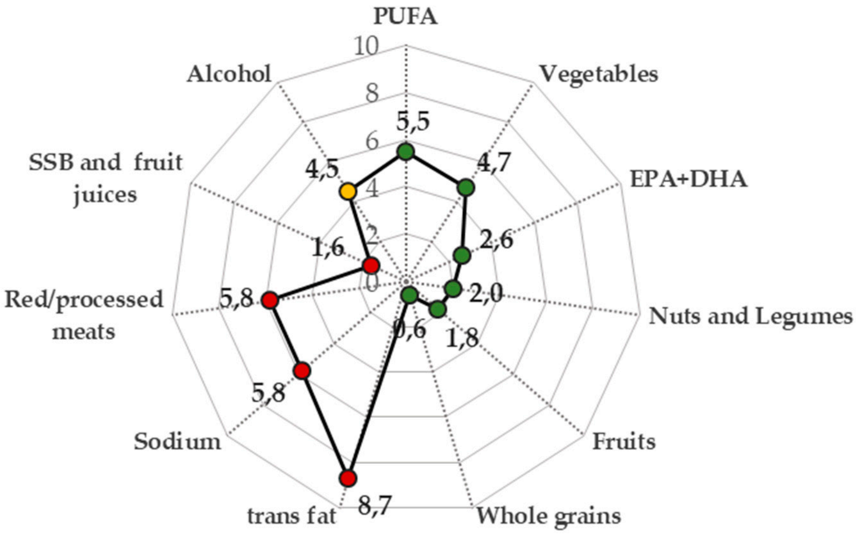

Supplemental Figure S1. Graphical description of Alternate Healthy Eating Index 2010 components. Components are ordered from highest to lowest mean AHEI-2010 scores separated by those with direct (green dots), indirect (red dots) and nonlinear (yellow dot) relation with the intake. SSB, sugar-sweetened beverages.
